# Patient-reported outcomes regarding the use of complementary and alternative medicine (CAM) in BCR::ABL1-negative myeloproliferative neoplasias

**DOI:** 10.1007/s00432-025-06421-5

**Published:** 2026-01-20

**Authors:** Carl C. Crodel, Charlotte Kohnle, Markus P. Radsak, Holger Nückel, Stefanie Jilg, Andreas Hochhaus, Florian H. Heidel, Jutta Hübner

**Affiliations:** 1 Klinik für Innere Medizin 2, Hämatologie und Onkologie, Universitätsklinikum Jena, Am Klinikum 1, 07745 Jena, Germany; 2 III. Medizinische Klinik, Universitätsklinikum Mainz, Mainz, Germany; 3 Gemeinschaftspraxis Prof. Dr. Holger Nückel/Dr. Johannes Matschke, Bochum, Germany; 4 Onkologie Erding, Erding, Germany; 5 Department of Medicine III, Klinikum rechts der Isar, Technische Universität München, Munich, Germany; 6 Hematology, Hemostasis, Oncology and Stem Cell Transplantation, Hannover Medical School (MHH), Hannover, Germany

**Keywords:** Myeloproliferative neoplasia (MPN), Complementary and alternative medicine (CAM), Polycythemia vera (PV), Essential thrombocythemia (ET), Myelofibrosis (MF)

## Abstract

**Introduction:**

BCR::ABL1-negative myeloproliferative neoplasms are chronic diseases characterized by high symptom burden due to systemic inflammation. Treatment objectives include cytoreduction and alleviation of symptoms. Besides standard therapies, complementary and alternative medicine (CAM) methods are frequently used and requested by cancer patients. Aim of this study was to assess the interest in and use of CAM by patients diagnosed with MPN.

**Methods:**

This study was conducted as a patient-reported, paper-and-pencil-based questionnaire.

**Results:**

166 patients with MPN were included, 72 (43.4%) male and 94 (56.6%) female. Median age was 65.0 years. Diagnoses included ET 66/166 (39.8%), PV 40/166 (24.1%), MF 51/166 (30.7%) MPN-U 8/166 (4.8%) and SM-AHN 1/166 (0.6%). Overall, more frequent use of CAM was documented in females (59.6%) compared to males (41.7%), *p* = 0.022. A significant proportion of patients reported on the ingestion of nutritional supplements: 44/163 (26.5%) vitamin D, 26/165 (15.7%) vitamin C, 19/165 (11.4%) zinc, 13/165 (7.8%) secondary plant products, 12/165 (7.2%) selenium, and 19/165 (11.4%) multivitamin preparations. Regarding other CAM-related measures: 5/164 (3.0%) used amygdalin, 4/164 (2.4%) mistletoe therapy, 11/165 (6.6%) acupuncture, 6/165 (3.6%) homeopathy, 11/165 (6.6%) yoga, 10/165 (6%) reported receiving spiritual support, while 3/165 (1.8%) used the services of “healers”. A higher rate of CAM use was found among patients with longer disease duration.

**Conclusions:**

Use of CAM was recorded in the majority of patients with MPN. Higher use of CAM-related measures was reported by women and patients with longer disease duration. Patients should be regularly consulted about the use of CAM, its risks should be considered and pointed out, and safe methods should be recommended.

**Supplementary Information:**

The online version contains supplementary material available at 10.1007/s00432-025-06421-5.

## Introduction

BCR::ABL1-negative myeloproliferative neoplasms (MPN) encompass a group of chronic hematologic cancers, including essential thrombocythemia (ET), polycythemia vera (PV), and primary myelofibrosis (PMF), characterized by clonal proliferation of hematopoietic stem cells and a high burden of symptoms that worsen over time (Palandri et al. 2018, 2025; Mesa et al. 2016; Harrison et al. 2017a; Manz et al. 2024). The pathogenesis of these MPN is complex, involving genetic, cytogenetic, and epigenetic aberrations that lead to dysregulated signaling pathways, chronic inflammation, and an increased risk of thrombosis and transformation to secondary acute myeloid leukemia (AML) (Perner et al. 2019). This complexity, driven by somatic mutations in key genes such as *JAK2*, *CALR*, and *MPL*, results in significant variability in disease presentation, progression, and treatment response (Harrison et al. 2017a; Mesa et al. 2018).

The mutational landscape in MPN is central to the understanding of disease progression and patient prognosis (Perner et al. 2025; Ling et al. 2025). The most common driver mutations, such as *JAK2V617F* and *CALR* exon 9 mutations, not only initiate clonal hematopoiesis but also significantly impact the inflammatory milieu that drives disease progression and symptom burden. These mutations, however, do not always act alone. Studies show that additional mutations in epigenetic regulators (*TET2*, *ASXL1*), splicing factors (*SRSF2*, *SF3B1*), and other genes linked to myeloid malignancies increase the risk of disease progression (Ling et al. 2025) and may dictate the rate and direction of clonal evolution (Palmer et al. 2019).

In MPN pathogenesis, the timing and sequence of mutation acquisition have critical implications for disease phenotype. For instance, the *JAK2V617F* mutation as an initial event commonly leads to PV with a high risk of thromboembolic complications, while as a secondary mutation, it often results in ET with a more indolent course (Harrison et al. 2013). These mutations drive abnormal cell proliferation while maintaining sufficient genomic stability, enabling long disease latency punctuated by periodic progression to more aggressive disease phases. The accumulation of somatic mutations, especially in PMF, correlates with clonal complexity, higher inflammatory markers, and inferior survival rates, with the number and type of co-occurring mutations being significant predictors of transformation risk.

A hallmark of MPN pathophysiology is chronic inflammation, which exacerbates both disease symptoms and progression. Elevated levels of inflammatory cytokines such as IL-6, TNF-α, and IL-8 have been detected in MPN patients, contributing to the pro-thrombotic state and marrow fibrosis characteristic of advanced disease phases (Verstovsek et al. 2022). This inflammatory environment promotes further genetic instability, providing a “fertile ground” for additional mutations, and fuels clonal expansion through paracrine signaling (Koschmieder et al. 2023). Chronic inflammation also influences symptomatology (Mesa et al. 2007), with patients frequently experiencing fatigue, pruritus, and constitutional symptoms that collectively diminish quality of life.

Despite advances in MPN therapy, current treatment options often fail to fully address disease progression and symptom burden. Conventional therapies, such as JAK inhibitors (e.g., ruxolitinib), improve symptom control and spleen size but do not consistently eliminate malignant clones or halt progression. Interferon therapies have shown potential in reducing clonal burden in early-phase MPNs, yet their effectiveness diminishes as the disease progresses and clonal complexity increases (Mesa et al. 2015). Given these limitations, patients frequently explore complementary and alternative medicine (CAM) to achieve symptom relief and psychological empowerment in managing their disease. CAM approaches, including herbal supplements, acupuncture, yoga, and meditation, aim to enhance quality of life by alleviating therapy-related side effects and promoting a sense of control in recovery.

While CAM offers supportive benefits, certain supplements and therapies may interact adversely with conventional treatments. Substances like amygdalin and herbal remedies containing St. John’s wort can alter drug metabolism through the cytochrome P450 enzyme system, impacting the efficacy of treatments such as imatinib and immunosuppressants (Dürr et al. 2000; Frye et al. 2004; Chan et al. 2023). Moreover, CAM practices lack robust clinical evidence, with some substances potentially posing significant health risks, such as hydrogen cyanide toxicity from amygdalin metabolism (He et al. 2020). Therefore, careful patient education and monitoring are essential to ensure safe integration of CAM into MPN management.

This study aims to explore the integration of CAM into MPN treatment regimens, focusing on patient interest, usage patterns, and the impact on quality of life. By understanding CAM’s role in MPN care, this work will establish a foundation for optimized patient-centered therapeutic strategies, balancing effective conventional treatment with evidence-based complementary approaches.

### Aims and objectives

Primary goal of this study was to generate evidence for the interest and use of complementary and alternative medicine (CAM) as a patient-reported outcome measurement in BCR::ABL1-negative MPNs. Secondly, our objective was to investigate various complementary and alternative medicine (CAM) methods and to identify distinct patterns in their use across different types of myeloproliferative neoplasms (MPNs). This approach aimed to highlight variations in CAM preferences and utilization among patients with distinct MPN subtypes, providing insights into how specific treatments might support unique symptom profiles and patient needs within each MPN category.

## Patients and methods

### Recruitment of participants

Recruiting centers have been identified through personal contact and email in Germany. The survey was conducted as a paper-and-pencil based questionnaire that was completed by the patient and finally approved by the responsible physician. The trial included 2 academic centers and 2 private hematology practices in Germany and recruited between September 2020 and October 2021. Identification of participants/MPN patients was conducted during regular outpatient visits.

### Statistics

Statistical analyses were performed with SPSS (version 25). Participating centers received no financial compensation for their contributions.

Descriptive analysis was used to assess the subtype of MPN, the type of driver mutation, therapy, interest, use and type of CAM, as well as the assessment of patient medication. Statistical correlations were conducted using a chi-square test. *P* value of < 0.05 was considered significant. All patients reported independent of duration, subtype of disease and medication. To simplify the descriptive analysis, the scales from the questionnaire were simplified.

### Questionnaires

The questionnaire contained questions on (1) patient characteristics, (2) current therapies and medications, (3) current laboratory values and molecular data, (4) disease related symptoms and (5) current use of CAM methods (Questionnaire as Supplement). For this part of the survey, we used a standardized questionnaire developed by the working group Prevention and Integrative Oncology of the German Cancer Society, which has been used in more than twenty studies on a broad ragen of cancer entities (Huebner et al. 2014). Therefore we addressed Patients’ need for information on disease and therapy, satisfaction with information and interest in and use of CAM-methods.

### Diagnosis, response criteria and risk scores

Diagnosis of MPN was confirmed at the participating centers according to the 2008 or 2016 WHO classifications of myeloid neoplasms (Arber et al. 2016; Barbui et al. 2018) depending on the date of diagnosis. The use of ELN criteria was recommended to assess for disease progression (Barosi et al. 2010).

### Ethics, consent and permissions

Data collection was done by every center. Data transmission was pseudonymized. Questionnaires and study materials were reviewed and approved by the institutional review board (Registration: 2020-1929-Bef).

## Results

### Patient characteristics

#### Gender, age and disease

In total, questionnaires from 166 MPN patients were collected. Gender distribution was balanced with 56.6% (*n* = 94) female and 43.4% (*n* = 72) male patients (Table 1). More than half of the patients (70.5%) had been diagnosed more than 3 years prior to the survey. Median age at the time of reporting was 62.3 years and median age at primary diagnosis was 55.3 years. Overall, the patient population was comparable to those investigated in large multicenter trials (Harrison et al. 2017b, c; Verstovsek et al. 2012).

**Table 1:** Patient characteristics

Characteristics	%	Total *n* = 166
*Sex*		
Male	43.4	72
Female	56.6	94
*Subtype*		
Polycythemia vera	24.1	40
Essential thrombocythemia	39.8	66
Myelofibrosis	30.7	51
MPN-U	4.8	8
Systemic mastocytosis with associated hematologic neoplasm (PV)	0.6	1
*Driver mutation*		
JAK2	70.9	117
CALR Exon 9	21.8	36
MPLW515	2.4	4
Triple negative	4.9	8
*MPN specific therapy*		
Ruxolitinib	37.3	62
Hydroxycarbamide	28.3	47
Interferon alpha	12.7	21
Watch and wait	10.8	18
Combination therapies/clinical trials	5.4	9
Allogeneic stem cell transplantation	2.4	4
Phlebotomy	1.8	3
Anagrelide	1.2	2

MPN subtypes comprised polycythemia vera (40/166, 24.1%), essential thrombocythemia (66/166, 39.8%), myelofibrosis (51/166, 30.7%), unclassifiable MPN (8/166, 4.8%) and one patient diagnosed with systemic mastocytosis with associated hematological neoplasm (SM-AHN with associated PV) (0.6%) (Table 1). Driver mutations are also shown in Table 1.

#### Treatment of MPN

The majority of patients received JAK1/2-inhibitor therapy with ruxolitinib (62/166, 37.3%). Hydroxyurea (HU) was used by 47/166 for cytoreductive therapy (28.3%). Other treatment options included interferon alpha (INF) monotherapy (21/166; 12.7%), or combinations with JAK-inhibitors (9/166, 5.4%) and anagrelide (2/166, 1.2%). 18/166 patients (10.8%) did not receive any specific therapy (watch and wait) while 3 PV patients (1.8%) remained on phlebotomy and 4/166 (2.4%) underwent allogenic stem cell transplantation (alloSCT). Treatment varied across cohorts due to individual drug approvals. In the MF cohort, the use of ruxolitinib, including in combination therapy, was most frequent (36/51; 70.6%), followed by hydroxyurea (HU) and watch-and-wait strategies (each 5/51; 9.8%), allogeneic stem cell transplantation (4/51; 7.8%), and interferon (1/51; 2.0%). In the PV cohort, most patients received either ruxolitinib (19/40; 47.5%) or HU (15/40; 37.5%), followed by interferon (3/40; 7.5%) and phlebotomy (3/40; 7.5%). In the ET cohort, HU was used most frequently (26/66; 39.4%), followed by interferon (18/66; 27.3%) and ruxolitinib (9/66; 13.6%). One patient received anagrelide monotherapy (1/66; 1.5%) and one patient received combination therapy with HU and anagrelide (1/66; 1.5%).

#### Cardiovascular risk-factors

Additional cardiovascular (CV) risk-factors were reported by nearly half of the patients (45.8%, 76/166), whereas 88/166 patients (53.0%) had no additional risk factors and in 1.2% (2/166) of cases no data was reported. Arterial hypertension was the most prevalent CV risk factor with 36.0% (59/164), followed by smoking 14.5% (24/164), diabetes mellitus 7.3% (12/164) and hypercholesterolemia 6.7% (11/164). Most common complications of MPN are thromboembolic events. At the time of data collection, 26.6% (41/154) of patients had already experienced a thromboembolic complication.

#### Educational status

The educational status was considered as the highest degree reached and indicated by the respective patient. With 66.5% (109/164), most patients had a secondary school degree, followed by a university degree in 25.0% (41/164) of cases. The remaining patients had either high school diplomas 6.7% (11/164) or no diploma 1.8% (3/164). Regarding their family status 74.1% (120/162) of the participants lived with a partner. 22.2% (36/162) lived alone and 3.7% (6/162) had other status.

### Patients’ needs for information and source of information

#### Patients’ needs for information on disease and therapy

Findings revealed that patients have a high need for information, with 94% (156/161) rating information on the disease itself as very or somewhat important. This demand was similarly strong for information on disease progression (97%, 159/164), treatment options (98.1%, 156/159), medication effects (98.1%, 157/160), and potential side effects (96.3%, 154/160).

Interest in self-help group resources was moderate, with only 56.1% (88/157) of patients considering it important, whereas 73.5% (122/156) deemed information on complementary and alternative medicine (CAM) essential.

Overall, patient satisfaction with information on disease and treatment was generally high, with 77.1%. 76.5% (101/132) of the patients were satisfied with information on self-help groups.

#### Source of information about disease, treatment and side effects

Information about “what is cancer” patients primarly get from their oncologist (69.7%, 115/166), internet (26.7%, 44/166), other media (14.5%, 24/166), general physician (16.4%, 27/166) and family and friends (9.7%, 16/166). Only two (1.2%) patients asked an alternative practitioner.

Information about their diagnosis they get from their oncologist (89.1%, 147/166), internet (20.6%, 34/166), general physician (18.8%, 31/166) other media (5.5%, 9/166) and family and friends (3.6%, 6/166). Only one patient (0.6%, 1/166) got information an alternative practitioner.

Primary information source about their therapy was their oncologist (89.1%, 147/166). Additional source include internet (12.7%, 21/166), general physician (5.5%, 9/166), other media (3%, 5/166), family and friends (1.8%, 3/166) and alternative practitioner 0.6%, 1/166).

When asked about side effects of their therapy most patient get the information from the oncologist (80.6%, 133/166). Less patients inform their self on the internet (12.1%, 20/166), by the general practitioner (4.8%, 8/166) and other media (3%, 5/166). Only one (0,6%, 1/166) patient claims to receive information from an alternative practitioner and one (0,6%, 1/166) from family and friends.

#### Source of information about CAM

Patients get from the oncologist (27.3%, 45/166), the internet 14.5% (24/166), other media 9.7% (16/166), family and friends 9.1% (15/166), alternative practitioner 5.5% (9/166) and general practitioner 4.2% (7/166).

### Interest in CAM-methods

Among the respondents, 45.1% (69/153) expressed an interest in CAM. Notably, of those interested, 56.5% (39/69) already had an interest in CAM prior to their cancer diagnosis, while 44.5% (30/69) developed interest following their diagnosis. A significant proportion of patients (28.8%, 44/153) reported no interest in CAM, and 26.1% (40/153) were uncertain.

Demographic analysis indicated gender-based differences, with a higher interest among female patients. Males showed lower interest in CAM (39.4%, *n* = 26/66). The average age of CAM-interested patients was younger, at 59.6 ± 14.1 years, compared to non-interested patients (63.5 ± 13.85 years). Statistically there was no significant correlation (*p* = 0.306).

**Fig. 1:**
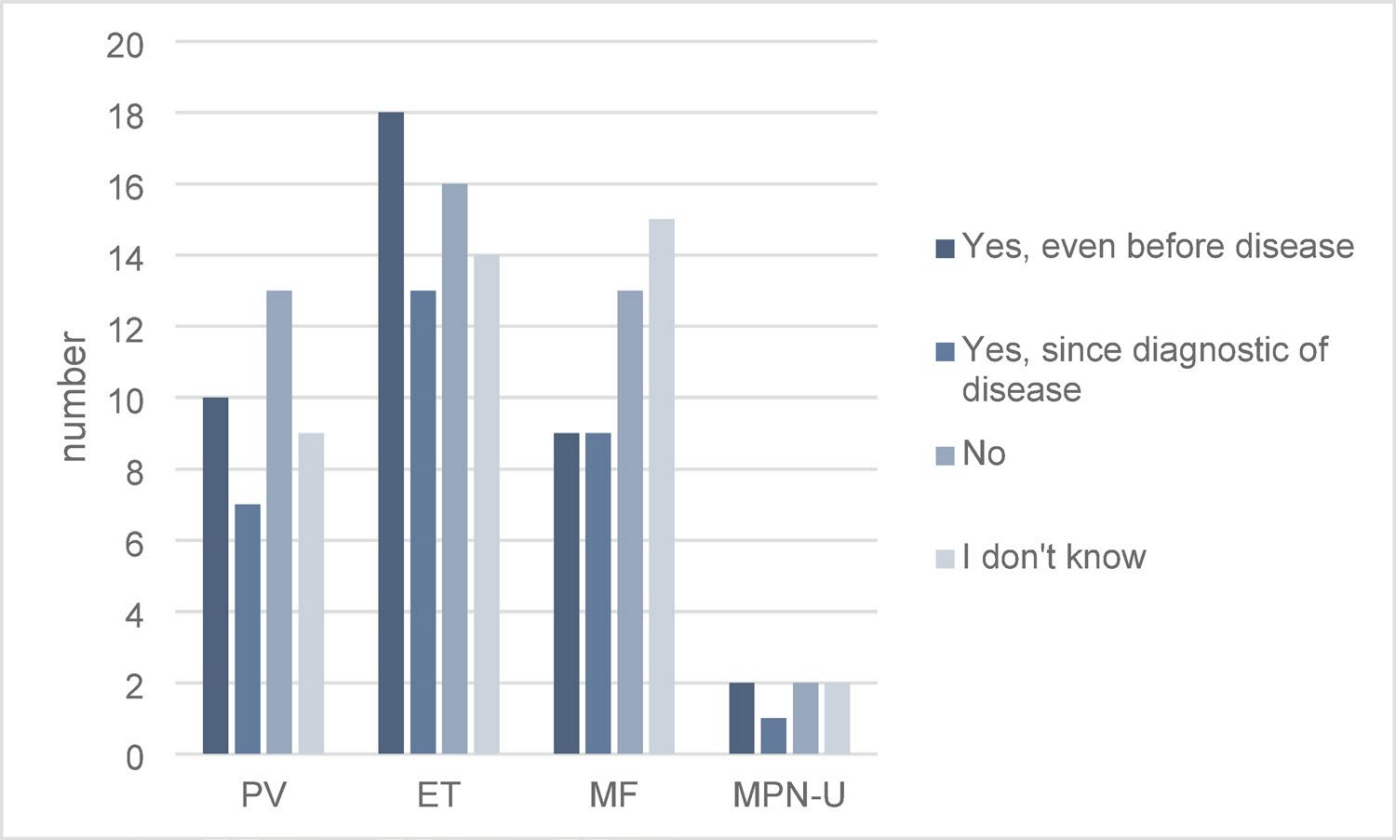
Interest in CAM in connection with the underlying disease (*N* = 153). *PV* polycythemia vera, *ET* essential thrombocythemia, *MF* myelofibrosis, *MPN-U* unclassifiable MPN

**Fig. 2:**
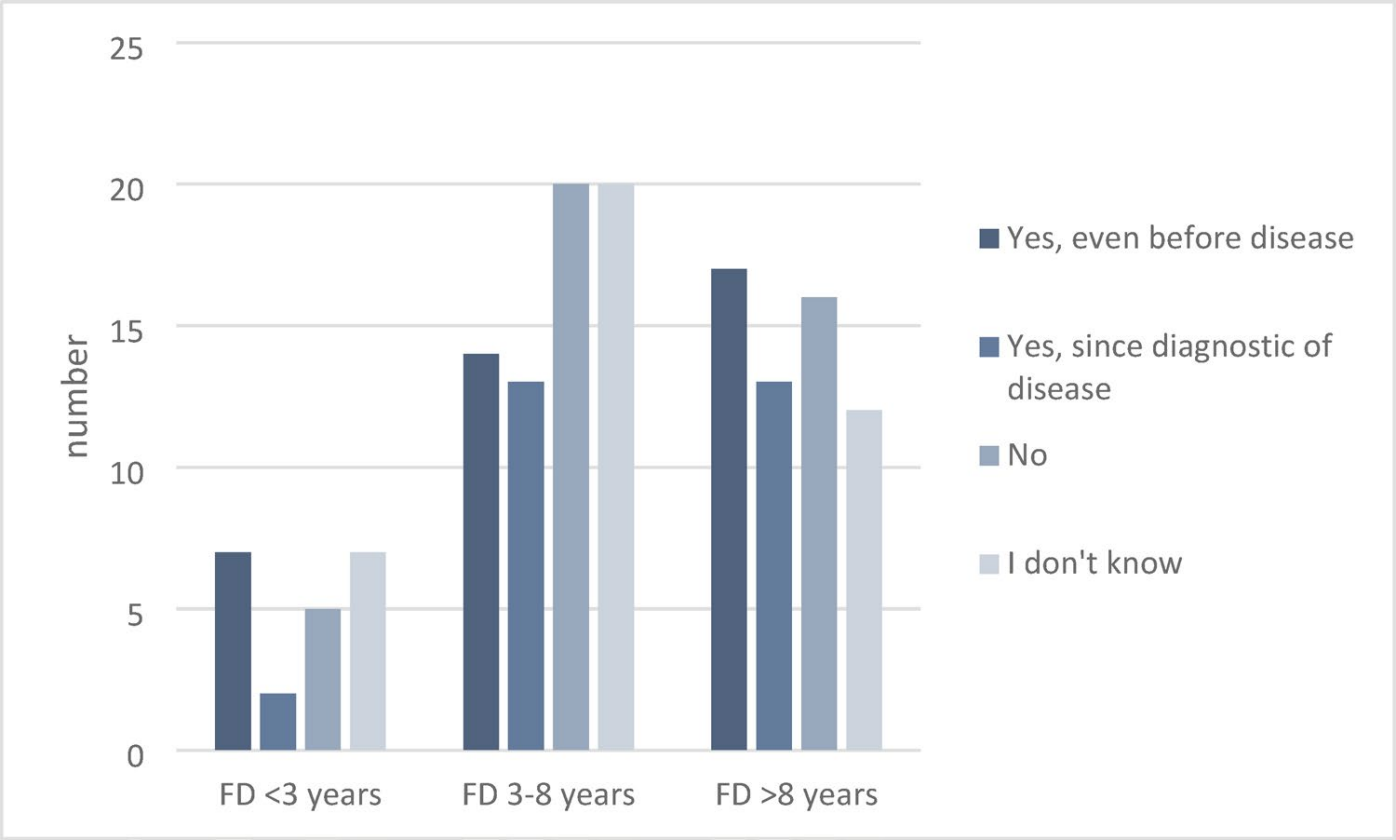
Interest in CAM in connection with the duration of the disease (*N* = 146), FD = first diagnosis

Analysis by MPN subtype: 47.0% (31/61) of patients with essential thrombocythemia (ET) reported interest, while myelofibrosis (MF) patients demonstrated a lower interest at 39.1% (18/46) (Fig. 1). Interest in CAM appeared to increase with disease duration, though this was not statistically significant (*p* = 0.64) (Fig. 2).

Educational background also played a role, as those with higher education, such as university degrees, showed a greater interest in CAM (64.1%, 25/39) compared to those with secondary school diplomas (39.6%, 40/101), highlighting educational influence on health behavior preferences.

### Use of CAM-methods

In this study, 51.8% (86/166) patients reported using a complementary and alternative medicine (CAM) method. Expectedly, CAM utilization was higher among those who expressed an interest in it, with 82.6% (57/69) of interested patients using a CAM method. Notably, 29.8% (25/84) of patients uninterested in CAM still reported CAM use.

Of the 19 complementary methods listed in the questionnaire, dietary supplements such as vitamins and trace elements were most commonly used. Of these, vitamin D was most frequently used by 27% of the patients. 15.6% (26/166) patients used vitamin C, 11.4% (19/166) zinc, 7.8% (13/166) phytochemicals, 7.2% (12/166) selenium and 11.4% (19/166) combinations of different vitamins.

3.0% (5/166) used apricot kernels/vitamin B17/amygdalin, which is based on prussic acid. A smaller proportion of patients resorted to mistletoe therapy 3.6% (6/166) or homeopathy. 6.6% (11/166) practiced yoga, Tai Chi or Qi Gong. A healer was visited by 1.8% (3/166) patients. 6% (10/166) patients performed prayers. 6.6% (11/166) followed a dietary form of nutrition (ketogenic diet, fast, vegan nutrition) (Table 2).

**Table 2:** Methods of CAM (*n* = 166)

CAM methods	*n*	%
Vitamin D	45/166	27
Vitamin C	26/166	15.6
Selenium	12/166	7.2
Zinc	19/166	11.4
Vitamin combinations	19/166	11.4
Phytochemicals	13/166	7.8
Acupuncture	11/166	6.6
Yoga/Tai Chi/Chi Gong	11/166	6.6
Diet	11/166	6.6
Prayer	10/166	6.0
Chinese herbs/tea	7/166	4.2
Homeopathy	6/166	3.6
Mistletoe	6/166	3.6
Vitamin B17/apricot kernels	5/166	3.0
Healer	3/166	1.8

In certain cases, patients reported using multiple complementary and alternative medicine (CAM) methods. Of the 51.8% (86/166) who used CAM, most utilized two methods, while use of five or more methods was notably less common. Excluding the widespread use of vitamin D (commonly prescribed in conventional medicine), 44% (73/166) reported using at least one CAM method (Table 3).

**Table 3:** Number of used CAM methods (*n* = 166)

Number of CAM methods	*n*	%
1	32	19.4
2	24	14.5
3	9	5.5
4	13	7.9
5	4	2.4
6	2	1.2
7	1	0.6
8	1	0.6
0	80	48.2
Total	166	100

**Fig. 3:**
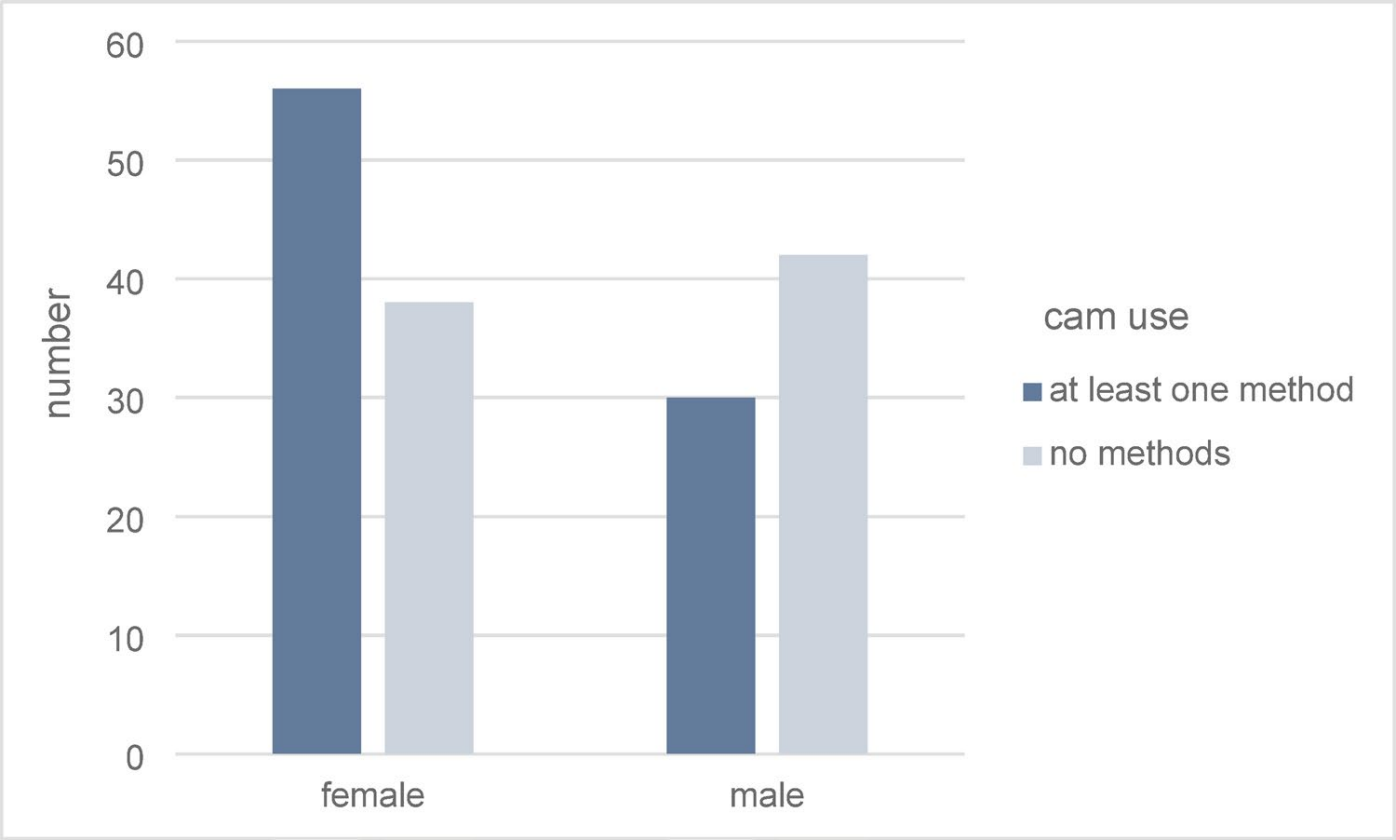
Application of CAM in relation to gender (*n* = 166)

Gender differences were observed: 59.6% (56/94) of female patients used CAM, compared to 41.7% (30/72) of male patients (*p* = 0.022) (Fig. 3). Disease type affected CAM preference as well, with essential thrombocythemia patients showing higher CAM use (65.2%) compared to myelofibrosis patients (37.2%), a difference that was statistically significant (*p* = 0.002). The average age of CAM users was slightly younger, at 60 ± 14.37 years, than that of non-users (64.4 ± 13.52 years). Patients with higher educational status showed an increased rate of CAM use. However, with a *p* value of 0.092, no significant correlation was found.

Focused on disease the usage of CAM was highest in patients with ET (43/66; 65.2%), followed by PV (22/40; 55%). The lowest rate on usage of CAM was observed in MF patients 19/51 (37.2%). There was no statistical significance (*p* > 0.05). Looking on MF patients more in detail 14/36 pts (38.9%), who received ruxolitinib also used CAM methods, while among the patients with other treatments 5/15 (33.3%) used CAM.

## Discussion

In recent years, complementary and alternative medicine (CAM) has gained prominence in routine medical practice, with usage rates in cancer patients rising from 25% in the 1970s–80s to 49% after 2000 (Horneber et al. 2012). Recent studies indicate usage rates between 52 and 95% (Rausch et al. 2011). However, CAM research specific to myeloproliferative neoplasms (MPN) remains scarce, prompting this study to explore MPN patients’ engagement with CAM.

In our cohort of 166 patients, approximately half reported using at least one CAM method (51.8%), and nearly half expressed general interest in CAM (45.1%). These findings align with usage rates reported in broader cancer populations. Patients reported a consistently high need for information regarding disease and therapy (Eisfeld et al. 2020), yet many indicated insufficient access to reliable guidance on CAM. This observation corresponds with previous research demonstrating that patients frequently do not discuss CAM use with their treating physicians, either due to lack of opportunity or concern about negative reactions (Robinson and McGrail 2004). Encouraging physicians to proactively address CAM may therefore improve communication, safety, and patient satisfaction.

Sociodemographic and disease-related patterns were evident in our study. Comparing these results with studies of cancer patients in general, CAM usage rates internationally range from 24 to 95% (Molassiotis et al. 2005), with averages around 40% (Horneber et al. 2012). In our study, MPN patients showed higher engagement with CAM (51.8%), even when excluding vitamin D, which is often prescribed. CAM use was more frequent among women and among patients with higher educational status, patterns consistent with other oncological settings.

Additionally, CAM interest and use were higher in ET patients (65.2%) compared to those with MF (37.2%) with statistical significance (*p* = 0.002), mirroring findings from previous research (Gowin et al. 2020). In our opinion, this is because MF patients are older and have often already taken or are taking several medications and may have less interest due to their advanced disease. Also the rate of ruxolitinib therapy was highest in this cohort. Due to the effectiveness of therapy, particularly in improving symptoms, it could be that this results in lower usage of CAM methods. Moreover, the duration of illness appeared to increase CAM engagement, especially in PV and ET, potentially as patients sought symptom management options beyond conventional treatments.

Even though it only affects a small proportion of the patients surveyed, we can see that the use of some CAM methods can be associated with significant risks. In the patient population, 3% used apricot kernels/amygdalin, whose mechanism is based on hydrocyanic acid. Study results show toxic effects in the body, so use poses a risk to patients (He et al. 2020).

Furthermore, dietary forms of nutrition that can lead to malnutrition and weight loss should be viewed critically. St. John’s wort was also used. Studies have shown that St. John’s wort influences the metabolism of various medications (Dürr et al. 2000). Taking St. John’s wort can lead to CYP induction, thereby increasing the breakdown of drugs and potentially reducing their effectiveness. Ruxolitinib is one of the drugs that is metabolized by the cytochrome P450 system.Our findings underscore a significant unmet demand for CAM information and support integration within conventional MPN care. Patients’ high need for CAM information and dissatisfaction with its current provision highlight the need for healthcare professionals to engage more actively in CAM discussions. Enhanced CAM training in medical curricula could support this goal, facilitating informed communication and a partnership-oriented approach in patient care (Astin et al. 2006). Expanding CAM research will further optimize patient-centered treatment strategies, allowing for safer, evidence-based integration of CAM into oncological care.

## Conclusion

This patient-reported outcome study on MPN patients focusing on their use of CAM is, to the best of our knowledge, the first assessment of this kind in Germany. Compared to the highly variable results of other studies, the use of CAM in the present study appears more balanced, with 45.1% of patients interested in CAM and more patients (51.8%) using it. It shows that CAM plays a significant role in the treatment of patients with MPN. One aspect frequently expressed by the participants is lack of information on CAM transported by physicians. This led to lack of satisfaction regarding patient information and underlines the current shortcomings in daily clinical practice.

Overall, our findings emphasize the importance of *systematically integrating CAM discussions into routine MPN care*. Key components include:

1. routinely asking about current CAM use,
2. providing balanced and evidence-based information,
3. recommending safe, supportive therapies (e.g., mind-body approaches, physical activity), and.
4. referring to established integrative oncology guidelines where available.

Thus, the topic CAM should be proactively addressed by MPN specialists. Increased awareness and further training of medical professionals in the field of CAM should be considered, as well as information for patients about the risks, side effects and possible (dis-)advantages of CAM. The use of the S3 guideline on complementary medicine in the treatment of oncology patients as a guide can be helpful to guide patient information and achieve patient awareness and satisfaction (including improved physician-patient relationship, communication and treatment success) (*S3-Leitlinie Leitlinienprogramm - Onkologie—Deutsche Krebsgesellschaft*,* Deutsche Krebshilfe*,* Awmf): KomplementäRmedizin in Der Behandlung Von Onkologischen Patientinnen*,* Langversion 1.0*,* 2021*,* Awmf Registernummer: 032/055ol*)

Further prospective studies are warranted to evaluate the efficacy and safety of specific CAM modalities in MPN and to better define their role in symptom and quality-of-life management. Establishing a universally accepted definition of complementary and alternative medicine (CAM), along with a clear classification of the methods it encompasses, is crucial. Such a standardized framework would enhance the comparability of studies by ensuring consistency in what is measured and reported. This approach would enable more accurate cross-study analysis, facilitating a clearer understanding of CAM’s role and effects across different patient populations and research contexts.

### Limitation

We consider our survey to be a pilot study and have placed emphasis on keeping the questionnaire short. There are currently no questionnaires for CAM that have been validated on large cohorts. But the choice of a paper–pencil based questionnaire can be a limitation of this work. Predefined answer options often make it easier for patients to answer questions, but harbor the risk of not finding an adequate answer option from their perspective. Free text answers offer more flexibility here but are associated with difficulties defining relevant endpoints. A definition bias could occur when asking about CAM methods, which could lead to potentially relevant methods not being mentioned. In two recent studies, significantly more CAM methods were explicitly listed in the questionnaire (Gowin et al. 2020; Rausch et al. 2011). It is therefore possible that the rates of CAM use in these two studies are higher than in our study. We have listed a selection of methods and offered patients the opportunity to specify further methods using free text. This may have limited reporting if patients did not use the free-text field. For future research, it would be advisable to provide a more comprehensive list of CAM methods. This could result in an even more detailed description of CAM use in MPN patients. For a more detailed analysis, it would be worthwhile in future to use either structured patient interviews or for better comparison with other studies, the questionnaires used there. In order to prove the positive benefits of CAM methods, future randomised studies are needed, particularly in the field of MPN diseases.

The data was collected retrospectively, which represents a snapshot of the immediate situation. Used CAM methods in the past, which already have been completed, were not queried. Repeated data collection could lead to fluctuations in the prevalences.

## Supplementary Information

Below is the link to the electronic supplementary material.

Supplementary Material 1

## Data Availability

No datasets were generated or analysed during the current study.
