# Supplementary material for: Patient-reported outcomes regarding the use of complementary and alternative medicine (CAM) in BCR::ABL1-negative myeloproliferative neoplasias
Source: J Cancer Res Clin Oncol. 2026 Jan 20;152(1):41. doi: 10.1007/s00432-025-06421-5 (PMC12819938; doi:10.1007/s00432-025-06421-5)
Supplement: Supplementary file 1 — Supplementary Material 1 [file 432_2025_6421_MOESM1_ESM.docx]

**Patient-reportet outcomes regarding the use of complementary and alternative medicine (CAM) in BCR::ABL1-negative myeloproliferative neoplasias**

**Supplemental Data**

Contents

1 Supplemental tables

Supplemental table 1 - Comorbidities and risk factors

Supplemental table 2 - Highest Educational status of patients

Supplemental table 3 - Relation ship status

2 Supplemental figures

Supplemental figure 1 - Interest in CAM by gender

Supplemental figure 2 - Interest in CAM in relation to educational status

Supplement figure 3 - Relationship between educational status and the use of CAM methods

Supplement figure 4 - Application of CAM methods for the different underlying diseases

3 Questionaire

**1 Supplemental tables**

Table 1 Comorbidities and risk factors in patients (n=164)

| ***Characteristics*** | ***%*** | ***Total n=164*** |
| --- | --- | --- |
| Thromboembolic Complications | 26.6 | 41 |
| Cardiovascular risk factors | 46.3 | 76 |
| Arterial hypertension | 36 | 59 |
| Smoking | 14.6 | 24 |
| Dyslipoproteinemia | 6.7 | 11 |
| Diabetes mellitus | 7.3 | 12 |
| Apoplex, Tansient ischemic attack | 9.1 | 15 |
| Heart attack | 3.7 | 6 |
| Heart failure | 3 | 5 |
| Coronary heart disease | 6.1 | 10 |
| Chronic obstructive pulmonary disease | 2.4 | 4 |
| Kidney failure | 6.1 | 10 |

Table 2 Highest Educational status of patients (N=164)

| ***Educational status*** | ***%*** | ***Total n=164*** |
| --- | --- | --- |
| No degree | 1.8 | 3 |
| Secondary school/Intermediate school leaving certificate | 66.5 | 109 |
| High school diploma | 6.7 | 11 |
| University/Universitys of applied science | 25 | 41 |

Table 3 Relation ship status (N=164)

| ***Relationship status*** | ***%*** | ***Total n=164*** |
| --- | --- | --- |
| Married | 64.8 | 105 |
| Living in a stable relationship | 9.3 | 15 |
| Single | 9.9 | 16 |
| Widowed | 12.3 | 20 |
| Other status | 3.7 | 6 |

**2 Supplemental figures**

Figure 1 - Interest in CAM by gender (N=153)

This figure shows the differences in interest in CAM depending on the gender of the patients. There is increased interest among female patients.

Figure 2 - Application of CAM methods in relation to educational status (N=152)

This figurese shows the Application of CAM methos in relation to the educational status of the patients. Overall, 68.3% (28/41) of patients with higher education used a CAM method, while 45.9% (50/109) of those with lower education did so. With a p-value of 0.092, no significant correlation was found.

Figure 3 - Relationship between educational status and the use of CAM methods (N=164)

This figure shows the relationship between educational status, use of CAM methods and in connection with the time of diagnosis of the disease. Patients with a higher level of education showed a significantly higher interest in CAM (p=0.007). The proportion of those was higher at 64.1% (25/39) than of those with lower education (39.6% (40/101). Furthermore, among respondents with a higher educational status, 68% of respondents showed this interest even before the cancer diagnosis. For patients with a lower educational status, no significant difference was found depending on whether they showed interest before or after their cancer diagnosis.

Figure 4 - Application of CAM methods for the different underlying diseases (N=166)

This figure shows the use of CAM methods depending on the underlying diese. It show tha the use of CAM methods in highest in patients with ET, followed by PV and lower in patients with MF or MPN-U.

**3 Questionaire**

**1. Fragen zu ihrer Person**

**1.1 Alter:** ○ unter 30 Jahre

○ 31-50 Jahre

○ 51-70 Jahre

○ 71-80 Jahre

○ > 80 Jahr

**1.2 Geschlecht:** ○ männlich ○ weiblich

**1.3 Schulausbildun**g**:** ○ kein Abschluss

○ Haupt-/Realschule oder vergleichbare mittlere Abschlüsse

○ Abitur

○ Studium/Fachhochschule

**1.4 Beziehungsstatus**

○ Verheiratet

○ in fester Partnerschaft lebend

○ ledig

○ verwitwet

○ sonstiger Welcher? ______________

**1.5 Welche Medikamente nehmen Sie ein?**

_____________________________________________________________________________________________________________________________________________________________________________________________________________________________________________________________________________________________________________________________________________________________________________________________________________________________________________________________________________________________________

**2. Die folgenden Fragen beziehen sich auf Informationen zu Ihre Krebserkrankung**

| **2.1. Wie wichtig sind Ihnen …** | **sehr wichtig** | **eher wichtig** | **eher unwichtig** | **sehr unwichtig** |
| --- | --- | --- | --- | --- |
| …Informationen zu Ihrer Erkrankung insgesamt? |  |  |  |  |
| … Informationen über weitere Unterstützungsmöglichkeiten (z.B. Selbsthilfegruppen, soziale Dienste)? |  |  |  |  |
| ... Informationen zum Verlauf Ihrer Erkrankung? |  |  |  |  |
| … Informationen über die Therapie Ihrer Erkrankung? |  |  |  |  |
| … Informationen über die Wirkung der verordneten Krebsmedikamente? |  |  |  |  |
| … Informationen über die Nebenwirkungen der Behandlung und ihre Therapie? |  |  |  |  |
| … Informationen zu Methoden der Naturheilkunde |  |  |  |  |
| **2.2. Wie zufrieden sind Sie mit ….** | **sehr zufrieden** | **eher zufrieden** | **eher unzufrieden** | **sehr unzufrieden** |
| … Informationen zu Ihrer Erkrankung insgesamt? |  |  |  |  |
| … Informationen über weitere Unterstützungsmöglichkeiten (z.B. Selbsthilfegruppen, soziale Dienste)? |  |  |  |  |
| ... Informationen zum Verlauf Ihrer Erkrankung? |  |  |  |  |
| … Informationen über die Therapie Ihrer Erkrankung? |  |  |  |  |
| … Informationen über die Wirkung der verordneten Krebsmedikamente? |  |  |  |  |
| … Informationen über die Nebenwirkungen der Behandlung und ihre Therapie? |  |  |  |  |
| … Informationen zu Methoden der Naturheilkunde |  |  |  |  |

**2.3. Woher haben Sie die Informationen zu Ihrer Krankheit und Therapie?**

|  | **Onkologe** | **Hausarzt** | **Heil-**  **praktiker** | **Familie Freunde** | **Internet** | **Andere Medien (TV, Radio, Bücher, Zeitschriften)** | **Andere und zwar** |
| --- | --- | --- | --- | --- | --- | --- | --- |
| Information, was Krebs ist |  |  |  |  |  |  |  |
| Information zu meiner Diagnose |  |  |  |  |  |  |  |
| Information zu meiner Therapie |  |  |  |  |  |  |  |
| Information zu den Nebenwirkungen meiner Therapie |  |  |  |  |  |  |  |
| Information dazu, was man gegen diese Nebenwirkungen tun kann |  |  |  |  |  |  |  |
| Information zu Methoden der Naturheilkunde |  |  |  |  |  |  |  |

**5. Die folgenden Aussagen können mehr oder weniger auf Sie zutreffen. Bitte geben Sie bei jeder Aussage an, inwieweit diese auf Sie persönlich zutrifft.**

|  | **Trifft gar nicht zu** | **Trifft wenig zu** | **Trifft etwas zu** | **Trifft ziemlich zu** | **Trifft voll und ganz zu** |
| --- | --- | --- | --- | --- | --- |
| In schwierigen Situationen kann ich mich auf meine Fähigkeiten verlassen |  |  |  |  |  |
| Die meisten Probleme kann ich aus eigener Kraft gut meistern |  |  |  |  |  |
| Auch anstrengende und komplizierte Aufgaben kann ich aus eigener Kraft gut lösen |  |  |  |  |  |

**6. Im Folgenden verwenden wir den Begriff Komplementäre Medizin als Oberbegriff zu allen unterstützenden Verfahren die Patienten selber machen (z.B. Nahrungsergänzungsmittel, Heilpflanzen, Homöopathie, Mistel, Entspannungsverfahren, Bewegung, Yoga etc.)**

**6.1. Interessieren Sie sich für Komplementäre Medizin?**

○ ja, schon vor der Tumorerkrankung

○ ja, aber erst seit der Tumorerkrankung

○ nein

○ ich weiß nicht

Wenn Sie die letzte Frage mit Ja beantwortet haben:

**6.2. Welche komplementären Methoden nutzen Sie? (Sie können mehrere Antworten ankreuzen.)**

| **Nahrungsergänzungsmittel** | Vitamin D |  |
| --- | --- | --- |
|  | Vitamin C |  |
|  | Selen |  |
|  | Zink |  |
|  | Kombination aus Vitaminen, Spurenelementen |  |
|  | Präparate aus sekundären Pflanzenstoffen (z.B. Curcumin, Lycopin…) |  |
| **Mistel** | |  |
| **Heilpflanzen** | Wenn ja, welche? |  |
| **Akupunktur** | |  |
| **Chinesische Kräuter / Tees** | |  |
| **Homöopathie** | |  |
| **Gebet** | |  |
| **Yoga / Tai Chi / Qi Gong** | |  |
| **Besuch bei einem Heiler** | |  |
| **Vitamin B17/Aprikosenkerne** | |  |
| **Besondere Ernährungsweisen** | sehr wenig Kohlenhydrate/ketogene Ernährung |  |
|  | Vegane Ernährung |  |
|  | Fasten |  |
| **Anderes (bitte eintragen)** |  |  |
|  |  |  |
|  |  |  |
|  |  |  |

**7. Bitte kreuzen Sie bei den folgenden Fragen die Zahl zwischen 1 und 7 an, die am besten auf Sie zutrifft**

1. **Wie würden Sie insgesamt Ihren Gesundheitszustand während der letzten Wochen einschätzen?**

1 2 3 4 5 6 7

Sehr schlecht ausgezeichnet

1. **Wie würden Sie insgesamt Ihre Lebensqualität während der letzten Woche einschätzen?**

1 2 3 4 5 6 7

Sehr schlecht ausgezeichnet

**8. Nachstehend finden Sie einige Aussagen, die Leute manchmal machen, wenn sie sich über ihre Gesundheit unterhalten. Bitte geben Sie an, wie stark diese Aussagen für Sie zutreffen.**

| **Aussagen** | **Stimmt nicht 1** | **Stimmt kaum 2** | **Stimmt eher 3** | **Stimmt genau 4** |
| --- | --- | --- | --- | --- |
| 1. Letzten Endes bin ich derjenige, der dafür verantwortlich ist, für meine Gesundheit zu sorgen. |  |  |  |  |
| 2 Das Wichtigste für meine Gesundheit ist, eine aktive Rolle in meiner Gesundheitsversorgung zu übernehmen. |  |  |  |  |
| 3. Ich bin überzeugt, dass ich selbst etwas unternehmen kann, um Krankheiten vorzubeugen. |  |  |  |  |
| 4. Ich weiß bei jedem meiner Medikamente, weshalb ich es nehme |  |  |  |  |
| 5. Ich bin überzeugt, zu wissen, wann ich zum Arzt gehen muss und wann ich ein Gesundheitsproblem selbst behandeln kann |  |  |  |  |
| 6. Ich bin überzeugt, dass ich meinem Hausarzt meine Sorgen mitteilen kann, auch wenn er mich nicht direkt darauf anspricht |  |  |  |  |
| 7. Ich bin überzeugt, dass ich die zu Hause notwendigen medizinischen Behandlungen selbst durchführen kann |  |  |  |  |
| 8. Ich kenne die Ursachen meiner Beschwerden. |  |  |  |  |
| 9 Ich kenne verschiedene Behandlungsoptionen für meine Erkrankungen |  |  |  |  |
| 10. Ich war bisher in der Lage, Veränderungen meiner Lebensgewohnheiten - wie gesunde Ernährung und Bewegung - aufrechtzuerhalten |  |  |  |  |
| 11. Ich weiß, wie ich einer Verschlechterung meines Gesundheitszustandes vorbeugen kann |  |  |  |  |
| 12. Ich bin überzeugt, Lösungen zu finden, wenn sich mein Gesundheitszustand verschlechtert |  |  |  |  |
| 13. Ich bin überzeugt, dass ich Veränderungen meiner Lebensgewohnheiten - wie Diät und körperliche Bewegung - auch in stressigen Zeiten fortführen kann. |  |  |  |  |

**8. Angst und Unsicherheit**:

8.1. Die folgenden Fragen beschäftigen sich damit wie häufig Sie sich in den letzten 12 Monaten durch Angst und Unsicherheit belastet gefühlt haben. In jeder Zeile bitte nur eine Antwort ankreuzen

|  | **Nie** | **Selten** | **Manchmal** | **häufig** | **Sehr oft** |
| --- | --- | --- | --- | --- | --- |
| Wenn ich an den weiteren Verlauf meiner Erkrankung denke, bekomme ich Angst. |  |  |  |  |  |
| Mich beunruhigt, was aus meiner Familie wird, wenn mir etwas passieren sollte. |  |  |  |  |  |
| Der Gedanke, ich könnte wegen Krankheit in der Arbeit ausfallen, beunruhigt mich. |  |  |  |  |  |
| Ich sehe ohne Hoffnung in die Zukunft. |  |  |  |  |  |
| Ich verbringe viel Zeit damit, mir Sorgen um meine Gesundheit zu machen. |  |  |  |  |  |
| Ich bin zuversichtlich, meine persönlichen Ziele zu verwirklichen |  |  |  |  |  |
| Entscheidungen, die ich treffe, kann ich später im Leben auch wieder ändern |  |  |  |  |  |
| Ich befürchte, dass ich durch die Krankheit vereinsame. |  |  |  |  |  |
| Ich habe Angst, durch meine Krankheit eines Tages anderen zur Last zu fallen. |  |  |  |  |  |
| Ich befürchte eines Tages aufgrund der Erkrankung pflegebedürftig zu werden. |  |  |  |  |  |
| Ich befürchte, andere Menschen nehmen mich nur noch als Kranke/n wahr. |  |  |  |  |  |
| *Bitte nur ausfüllen, wenn Sie in einer Partnerschaft leben…* | | | | | |
| Ich befürchte, dass meine Erkrankung unsere Beziehung belastet. |  |  |  |  |  |
| Ich habe Angst, dass mein Partner die Belastung durch meine Krankheit nicht aushält |  |  |  |  |  |
| Ich habe Angst, durch meine Krankheit von meinem Partner abhängig zu werden. |  |  |  |  |  |

**8.2. Auf einer Skala von 0 bis 10, kreisen Sie bitte die Zahl ein die am Besten beschreibt wie belastet sie sich in den letzten Wochen einschließlich heute gefühlt haben.**

**Gar nicht belastet** 🡪 **1 2 3 4 5 6 7 8 9 10 🡪 extrem belastet**
